# Predicting Length of Stay using machine learning for total joint replacements performed at a rural community hospital

**DOI:** 10.1371/journal.pone.0277479

**Published:** 2022-11-10

**Authors:** Srinivasan Sridhar, Bradley Whitaker, Amy Mouat-Hunter, Bernadette McCrory

**Affiliations:** 1 Mechanical and Industrial Engineering, Montana State University, Bozeman, Montana, United States of America; 2 Electrical and Computer Engineering, Montana State University, Bozeman, Montana, United States of America; 3 Bozeman Health, Bozeman, Montana, United States of America; Baqai Medical University, PAKISTAN

## Abstract

**Background:**

Predicting patient’s Length of Stay (LOS) before total joint replacement (TJR) surgery is vital for hospitals to optimally manage costs and resources. Many hospitals including in rural areas use publicly available models such as National Surgical Quality Improvement Program (NSQIP) calculator which, unfortunately, performs suboptimally when predicting LOS for TJR procedures.

**Objective:**

The objective of this research was to develop a Machine Learning (ML) model to predict LOS for TJR procedures performed at a Perioperative Surgical Home implemented rural community hospital for better accuracy and interpretation than the NSQIP calculator.

**Methods:**

A total of 158 TJR patients were collected and analyzed from a rural community hospital located in Montana. A random forest (RF) model was used to predict patient’s LOS. For interpretation, permuted feature importance and partial dependence plot methods were used to identify the important variables and their relationship with the LOS.

**Results:**

The root mean square error for the RF model (0.7) was lower than the NSQIP calculator (1.21). The five most important variables for predicting LOS were BMI, Duke Activity Status-Index, diabetes, patient’s household income, and patient’s age.

**Conclusion:**

This pilot study is the first of its kind to develop an ML model to predict LOS for TJR procedures that were performed at a small-scale rural community hospital. This pilot study contributes an approach for rural hospitals, making them more independent by developing their own predictions instead of relying on public models.

## Introduction

With increasing rates of Total Joint Procedures (TJR) in the United States (US), predicting length of stay (LOS) is vital for hospitals to control costs, manage resources, and prevent postoperative complications [1–3]. LOS is the period between the time when the patient is admitted after the surgery to the time when the patient is discharged from the hospital. A longer LOS has been shown to negatively affect the quality of care and patient satisfaction [4, 5]. Specifically, patients with longer LOS drastically increases surgical expenses due to the high average inpatient costs at hospitals, which is $2,000 to $3,000 per day [6–8]. Moreover, past studies have demonstrated that patients with longer LOS have higher chances of experiencing poor postoperative surgical outcomes such as readmission and discharge to nursing or rehabilitation facility [9, 10].

To better manage surgical costs, allocate resources, and improve patient satisfaction, researchers have identified factors responsible for longer LOS using various analytic tools, including statistical and machine learning (ML) models [2, 11–13]. However, limited work has been done to predict LOS at community hospitals located in rural areas. It is more challenging to predict patients’ LOS in rural than urban areas because community hospitals located in rural areas often lack adequate resources–such as data, staff, and expertise–needed to develop an accurate predictive model [14]. Instead, many hospitals use publicly available models that were developed to quantify patient risk before surgery [15].

One such available model is the National Surgical Quality Improvement Program (NSQIP) risk calculator. The NSQIP risk calculator is widely used by hospitals to predict risks for TJR procedures performed on knees, hips, and shoulders [15]. At a single hospital or institution, the NSQIP risk calculator can be useful for surgeons to assess patient risk, but it has been found to be suboptimal when predicting LOS for TJR procedures [8, 15, 16]. Interpretability is also a concern for the NSQIP risk calculator. In NSQIP, the risk factors are not quantified (i.e., it does not let the clinicians know which risk factor is most associated with a particular outcome). NSQIP’s lack of factors quantification demonstrates that this predictive tool may not be suitable for evidence-based decision-making patient optimization in PSH care models. Predicting LOS has become more vital for orthopedic clinicians since recently the Center for Medicare and Medicaid services (CMS) removed knee and hip arthroplasty from the inpatient list [17, 18]. Knowing the risk factors and which patient will stay longer in the hospital after surgery are pertinent metrics for clinicians, administrators, and payers to correctly evaluate resource utilization, cost, and severity of illness [19].

Recent research has explored a promising application of ML for predicting surgical outcomes [20]. ML is a part of Artificial Intelligence (AI) which uses algorithms to recognize patterns in data to make predictions [21]. In the past decade, the application of ML in healthcare increased drastically due to wider usage of Electronic Medical Record (EMR) systems, which enabled the generation of ‘big data’ [22–25]. Big data has motivated many researchers and clinicians to apply ML and predict various health outcomes to improve patient treatment and quality of care [26, 27]. Proportionally, the role of ML surged in the orthopedic field as well [20, 28, 29]. For example, Ramkumar et al. [30] developed an ML using a naïve Bayesian model to forecast LOS and payments for total hip arthroplasty (THA). The authors used it as a classification problem by grouping the inpatient payments to <$12000, $12100-$24000, and >$24000, and LOS to 1–2, 3–5, and 6+days. The ML model was found valid, reliable, and accurate in its predictions with AUC of 0.87 and 0.71 for LOS and payment respectively. Similarly, Li et al. [13] developed an ML model which accurately predicted the LOS for total knee arthroplasty (TKA) procedure with an AUC greater than 0.73. Gabriel et al. [31] predicted LOS using ML methods to determine patients who do not require prolonged LOS. The developed model was reliable with AUC of more than 0.7 and helped hospital administrators to plan resources to avoid both overcrowding and underutilization of TJR patients. Relatedly, Han et al. [32] predicted LOS for knee patients in China and identified that Random Forest model predicted more accurately than other ML models with AUC of 0.7. Aazad et al. [33] utilized various ML methods to predict the duration of surgery and LOS which significantly contributed to an increase in surgical cost. The study found that PyTorch MLP performed better among other ML models with least Mean Square Error of 0.89 and 0.69 for duration of surgery and LOS, respectively. Klemt et al. [34] used three ML methods to predict LOS for knee revision patients. The authors observed all three ML models performed well with AUC more than 0.8 and decision curve analysis with P-value <0.01. In addition, the authors identified that patients’ age, Charles comorbidity index, and body mass index, were strong predictors for predicting LOS. The above examples are some of the recent studies that used ML methods to predict LOS for TJR procedures. Several studies were also performed in predicting TJR outcomes including surgical site infection, readmission, discharge disposition, and other adverse events [20, 35].

Yet, limited research has been performed with ML in rural hospitals. In addition, to the authors’ knowledge, no research has been performed in predicting surgical outcomes at a rural hospital that adopted newly emerging coordinated surgical system in orthopedics—the Perioperative Surgical Home (PSH) [14]. The PSH model of care was created by the leaders within the American Society of Anesthesiologists (ASA) to improve surgical outcomes and patient experience [36–38]. Therefore, this research focuses on developing an ML model to predict LOS for TJR procedures performed at a PSH-implemented community hospital. Despite the challenges associated with limited availability of data, this study expects that the developed ML model will perform better in accuracy and interpretation than the NSQIP risk calculator.

## Methods

### Data collection and preprocessing

A rural community hospital formed an integrated PSH outpatient clinic in November 2018. The hospital was an 83-bed, licensed level-III trauma center primarily serving three rural counties, but often sees patients from more than 10 surrounding rural counties which span 9,000 square miles and approximately 136,000 residents. The PSH outpatient clinic was affiliated with the hospital began seeing patients preoperatively for TJR including hip, knee, and shoulder replacements. A written consent was obtained from the patients before their participation in this study. The consent was documented and attached to patient’s EMR for reference. The study had no patients who are younger than 18. The scope of this pilot study focused on elective procedures and excluded any revisions. A total of 158 TJR patients were analyzed retrospectively after visiting the PSH clinic during preoperative surgical assessment from August to December 2020. All preoperative surgical assessments were performed within 30 days before surgery.

A total of 28 independent variables were collected for each patient, which included 20 NSQIP preoperative characteristics and eight additional variables. The NSQIP characteristics were collected and entered manually into the risk calculator to determine NSQIP-predicted LOS [39]. Additional variables were extracted from the Electrical Medical Record (EMR) such as the Duke Activity Status Index (DASI) [40], living status (whether the patient was living alone or with family), patient’s household income, postoperative nausea and vomiting score (PONV) [41], depression (whether the patient was depressed at the time of the assessment), preoperative preparation period (the number of days between assessment and surgery), distance traveled by the patient (in miles, from their residence to the hospital), and patient primary insurance type (private or public payer). These additional variables were included in the analysis as they were found to be closely associated with risk for poor surgical outcomes in past studies [3, 37, 42–45]. After patient’s discharge from the hospital, the actual LOS was extracted from the EMR.

Eleven NSQIP categorical variables were excluded as there were no cases observed in those categories: steroid use, ascites, systemic sepsis, ventilator-dependent, emergency case, dissemination of cancer, congestive heart failure, chronic obstructive pulmonary disease, dyspnea, dialysis, and acute renal failure. After exclusion, a total of 17 variables were considered in the analysis (Table 1). The variable distance traveled by the patient was produced by calculating the mileage between their zipcode and the hospital on Google Maps [46]. There were no missing values for the independent variables except for patient’s household income. Thirty-six patients (23%) out of 158 declined to provide their household income to clinicians during the assessment. These missing values were imputed using the median income of the patient’s zipcode [47]. The 2019 US Census Bureau database was used to input the zipcode median income obtained from Montana Demographics by Cubits [48]. Additionally, the independent variables that were classified as ordinal were ranked accordingly for use in the correlation analysis (Table 1).

**Table 1:** Variable description.

	Variables	Type	Description
**Response Variable**	Length of Stay (LOS)	Continuous	Length of stay post surgery in hours
**NSQIP Variables [39]**	Procedure	Categorical	Total Knee (TKA), Total Hip (THA), Total Shoulder (TSA) Arthroplasty
	Age	Continuous	Patient age in years
	Sex	Dichotomous	Gender: Male, Female
	Functional Status	Ordinal	Fully Independent (1), Partially independent (2), Fully dependent (3)
	ASA Class	Ordinal	Healthy patient (1), Mild systemic disease (2), Severe systemic disease (3), Severe disease with constant threat to life (4)
	Diabetes	Dichotomous	No (1), Yes (2)
	Hypertension	Dichotomous	No (1), Yes (2)
	Current Smoker within 1 year	Dichotomous	No (1), Yes (2)
	BMI	Continuous	Body Mass Index in Kg/m^2^
**Additional variables**	Duke Activity Status Index [40]	Continuous	Functional capacity scaled from 2.74 (low functional activity) to 9.89 (high functional activity) in METs
	Living Status [45]	Dichotomous	Patient’s primary household status—lives alone, living with another
	Patient’s Household Income [42]	Ordinal	Patient’s household income level: <$49K (1), $50-99k (2), $100+k (3)
	Postoperative Nausea and Vomiting Score (PONV) [41]	Ordinal	To estimate nausea after surgery due to anesthesia, scaled 0 (low chances of nausea) to 4 (high chances of nausea)
	Depression [3]	Dichotomous	Depression at the time of preoperative assessment: No (1), Yes (2)
	Preoperative Engagement Period [49]	Discrete -Ordinal	Difference in time period (in days)
	Patient Insurance Type [50]	Dichotomous	Patient’s payer—Public, Private type
	Distance Travelled by the patient [44]	Continuous	Distance traveled by the patient from their resident to the hospital (in miles)

### Feature selection

Feature selection was performed to identify the most important features, i.e., independent variables to train a novel ML model, improve accuracy, and reduce computation time [51]. This pilot study used Spearman’s rank correlation filter method [52] and Boruta wrapper method [53] to identify the important independent variables to predict LOS.

#### Spearman’s rank correlation

**Fig 1:**
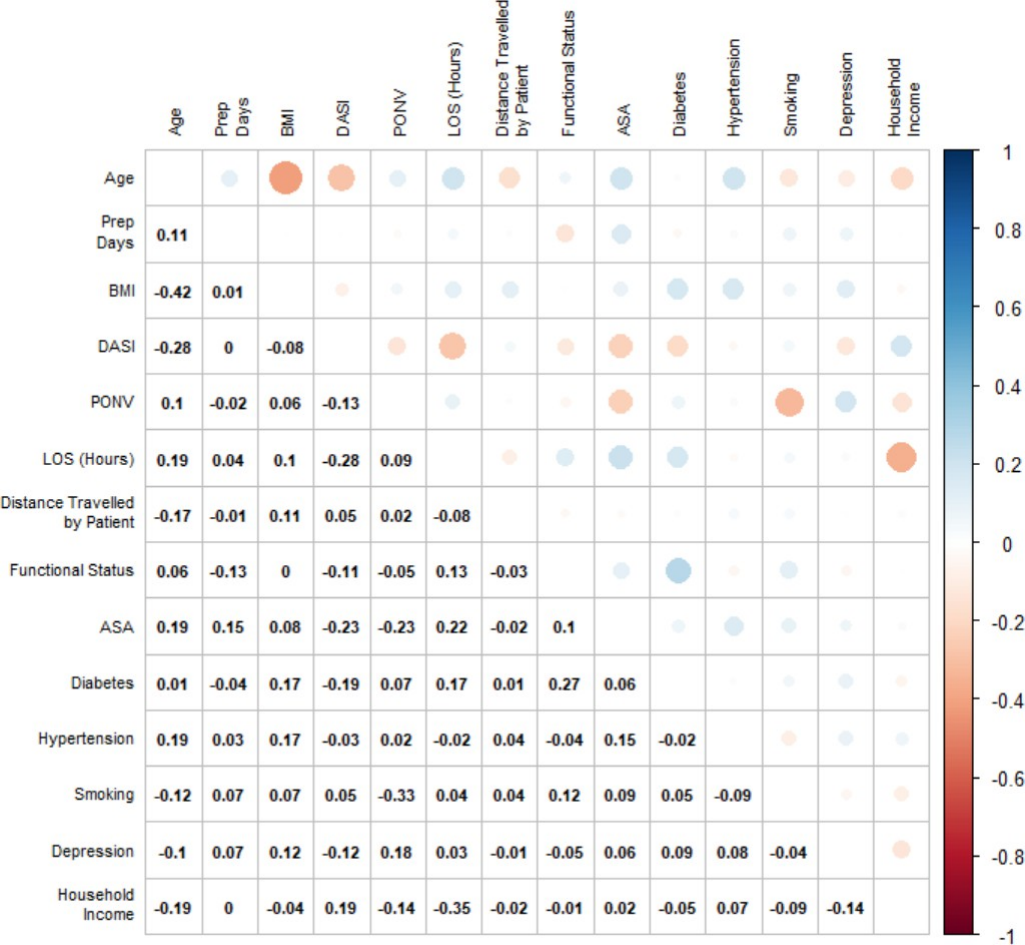
Correlation plot.

Correlation analysis was performed to identify highly correlated variables (correlation value close to 1 or -1). Independent variables that are highly correlated with one another can act as redundant in the analysis as they do not improve the model performance but increase the model complexity and computation time [54]. The database in this pilot study consisted of both continuous and ordinal variables and Spearman’s rank method was used to perform correlation analysis [52]. No highly correlated (correlation more than 0.7) independent variables were observed (Fig 1). Similarly, there were no highly correlated independent variables with dependent variable, LOS. The remaining feature selection was refined using the wrapper method.

#### Boruta feature selection method

Boruta feature selection is a wrapper method that utilizes the random forest algorithm to rank variable importance [53]. The Boruta uses shadow variables that are obtained by shuffling the original values across objects [53, 55]. The Boruta ranks variable importance using shadow variables as a reference. Any variable that showed higher importance than shadow variables is considered important [53]. The Boruta is known to have comparable, if not superior, ability in independent variable selection than other available feature selection methods [56].

**Fig 2:**
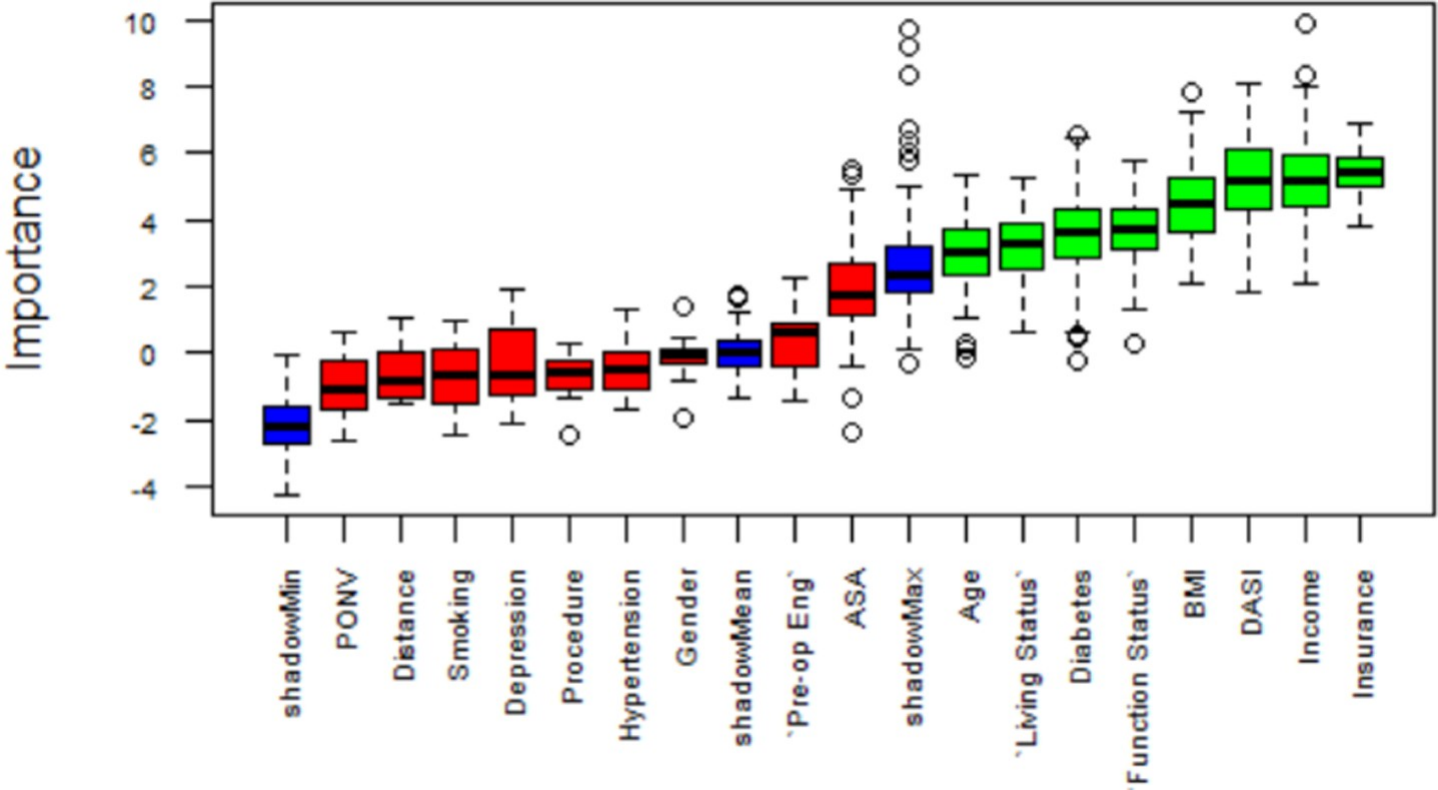
Independent variable selection using Boruta [53].

This study simulated Boruta for 100 runs to eliminate random errors in the results. The independent variables that were found important and statistically significant in the binomial distribution (P-value < 0.01) were selected for the prediction modeling. The variables that were found important were Insurance Type, Patient’s Household Income, DASI, BMI, Functional Status, Diabetes, Living Status, and Age (Fig 2). The rest of the independent variables were found not important. This study considered the important and excluded the non-important variables from the ML analysis.

### Model selection

The study found no strong linear correlations between dependent and independent variables indicating that the dependent variable and independent variables were non-linearly related. To identify non-linear patterns, Random Forest (RF) method was used. RF is an ensemble learning where the output of multiple decision trees is combined to deliver the final outcome or prediction [57]. Past studies also have exhibited the effectiveness of RF in predicting surgical outcomes with limited data, similar to this study [58, 59]. Compared to RF, other commonly known ML methods such as Neural Networks, Boosted Trees, and Support Vector Machines have more tuning parameters and often require more data to train [60–62]. Due to the very small sample size and RF being one of the most familiar ML methods in predicting orthopedic surgical outcomes [20], this preliminary study used only RF to predict patient LOS after TJR procedures performed at a community hospital. Other ML methods will be considered in the future upon more data availability (n> 2,000).

### Data splitting and tuning the parameter

The data was split into training (80%, n = 127) and testing (20%, n = 31) (Table 2). The data splitting and tuning were performed using the CARET package in R software [62].

**Table 2:** Baseline of training and test sample.

Variables		Train data (n = 127) Mean (SD) [Min, Max] or N (%)	Test Data (n = 31) Mean (SD) [Min, Max] or N (%)
Length of Stay (LOS)		41 (29) [3, 195]	36 (16.5) [5, 77.9]
Age		68.6 (9.63) [43, 91]	70.7 (7.43) [54, 84]
Functional Status			
	Independent	128 (98%)	31 (100%)
Diabetes			
	Yes	13 (10%)	2 (6%)
BMI		30.7 (7.25) [17, 58]	29.9 (4.28) [20, 40]
Duke Activity Score Index (DASI)		7.5 (1.67) [4.4, 9.89]	7.29 (1.5) [4.64, 9.89]
Living Status			
	Alone	26 (20%)	9 (29%)
Patient’s Household Income			
	<40K	50 (39%)	14 (45%)
	50-99k	56 (44%)	12 (39%)
Patient Insurance Type			
	Private	34 (27%)	5 (16%)

**Fig 3:**
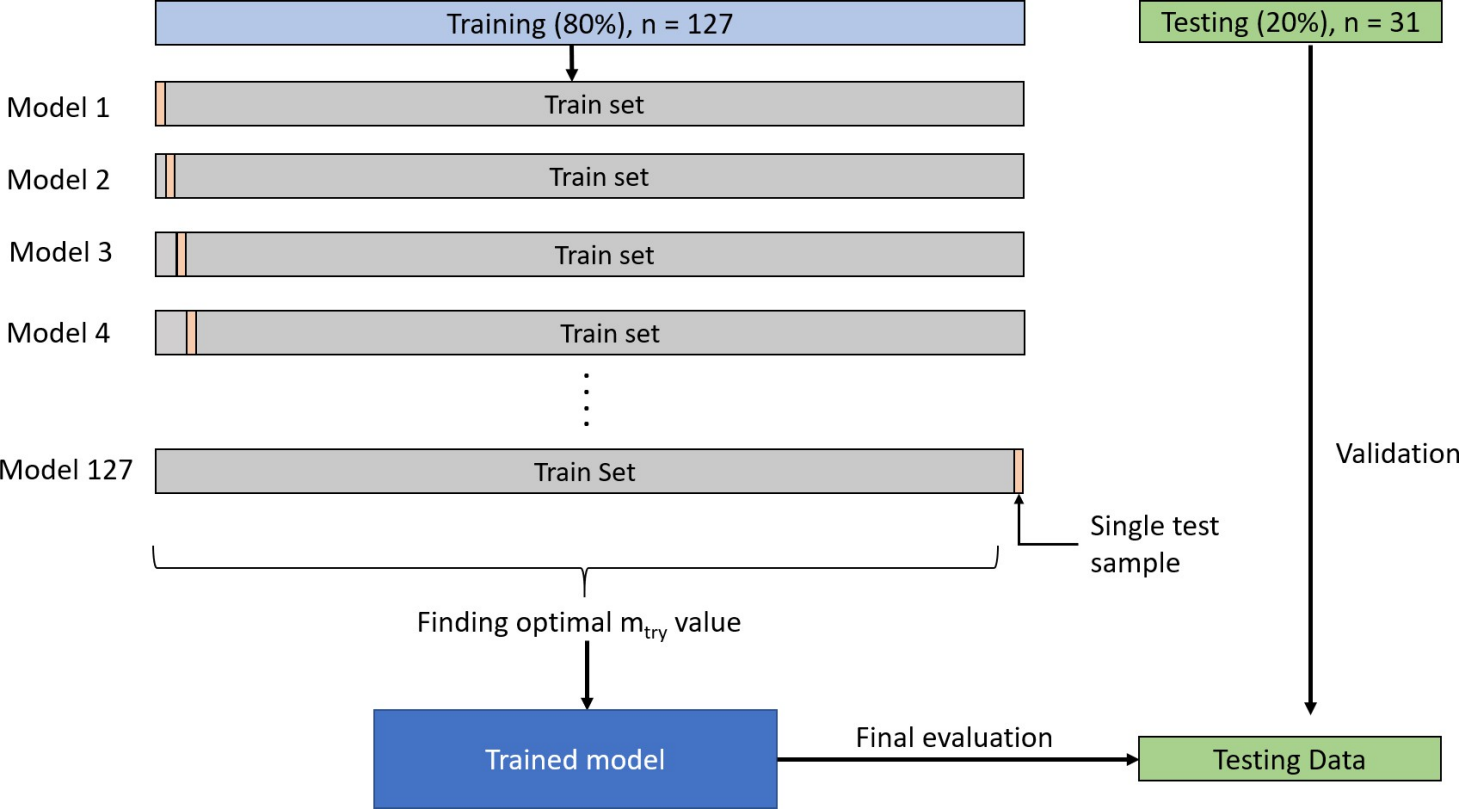
Leave One Out Cross Validation.

The RF has two main tuning parameters, which are the number of trees in the forest (n_tree_) and the number of variables randomly sampled as candidates at each split (m_try_). One thousand trees (n_tree_) were used in the forest, as recommended by past researchers [57, 62]. Having more trees in the RF does not affect the performance of the prediction negatively, but it can increase computation time [63]. The study expects no significant increase in computation time by using 1000 trees as compared to fewer trees (100 to 500 trees) because there were only 127 data points in the analysis. Leave One Out Cross Validation (LOOCV) was used to find the optimal number of variables available for splitting at each tree node–m_try_ [64] (Fig 3). The study chose LOOCV as it was more appropriate to use in smaller datasets [62, 65].

### Model validation

The NSQIP predicted LOS in days, segmented into half-day intervals. For appropriate comparison, the actual LOS and RF-predicted LOS, which were in hours, were converted to days. Conversion was performed in 12 hour-intervals, such that LOS less than or equal to 12 hours was considered 0.5 days, LOS less than or equal to 24 hours was considered 1 day, and LOS less than and equal to 36 hours was considered 1.5 days, and so on. The study was a regression problem as the response variable LOS was numeric and continuous. Therefore, the Root Mean Square Error (RMSE) was used as the cost function to validate the model’s prediction performance i.e., the lower the RMSE value, the better the model is performing [66]. Pearson’s correlation was also performed to determine the linear relationship between predicted LOS and actual LOS i.e., high correlation demonstrates better model performance. This pilot study expects the developed RF model to have a lower RMSE and higher correlation than NSQIP.

### Model interpretation

RF is an ensemble ML model that aggregates many independent decision trees to make a prediction. Though it greatly helps the accuracy of the prediction, the model acts as a black box and the interpretation is complex. In RF, each tree has a different structure as they are built based on the subset of predictors or independent variables that were randomly selected [57]. To understand and explain each tree in the forest is complex and nearly impossible, which makes the interpretability of a RF model difficult. However, past researchers have been able to interpret ML models (such as RF) substantially, if not completely, using different model-agnostic approaches [67, 68]. In ML, unlike model-specific methods, model-agnostic methods can be applied to any ML model for interpretation. This makes the model agnostic approaches more flexible and reliable than model-specific when interpreting different ML models [67]. This pilot study applied two widely used model-agnostic methods; permutation feature importance and partial dependence plots for interpreting and explicating the relationship of the variables in the RF model [69].

#### Permutation Feature Importance

Permutation Feature Importance (PFI) method measures importance by calculating the increase in prediction error after permuting the independent variable [70]. In other words, the independent variable in the data set is randomly permuted which degrades the relationship between that independent variable and the response variable. The importance of a variable is measured based on the difference in cost function before and after the variable is permuted [67]. The PFI approach uses randomness in the procedure to evaluate the importance. Thus, this study simulated this method for 100 times to minimize random errors and ranked the important variables based on the average value. The PFI algorithm used in this study adapted from [67, 71]:

Let j be the total number of independent variables
Let X be the independent variable
Let E be the baseline RMSE value for the trained model
Let F be a two-dimensional matrix of RMSE values after a feature is permuted in the training data
  1. For k = 1, 2, 3, ….100: (for simulating 100 times)
    - For i = 1, 2, 3, …‥j:
      - Permute the values of variable X_i_ in the training data.
      - Recompute the RMSE value on the permuted data–F_ki_.
  2. For i = 1, 2, 3, …‥j:
    - Compute average importance for each variable, Imp_i_ = $\frac{1}{100}\sum_{k=1}^{100}\left(E-F_{ki}\right)$
  3. Sort the average importance (Imp_i_) by descending order.

#### Partial Dependence Plot

The Partial Dependence Plot (PDP) is an another agnostic method that helps to understand the marginal effect of a variable on the predicted outcome of an ML model [72]. The PDP shows the relationship between a response variable and an independent variable whether they are linear, monotonic, or complex [67]. This demonstrates how the response variable changes as the value of an independent variable while considering the average effect of all the other independent variables in the model [69]. The biggest disadvantage of PDP is that it is effective when the variables are not correlated. However, this study had no strong correlation between any independent variables. Therefore, the PDP approach was more ideal method as they were easy to implement and more importantly, simple to interpret. In Eq 1, f_S_ was the partial function which was estimated by calculating the average value in the training data. The x_S_ was the independent variable that was being plotted in the PDP where S ⊂ (1, 2,…j). The ${\text{x}}_{\text{C}}^{\left(\text{i}\right)}$ were the rest of the independent variables in the training data where C was complement of S. The variable n was the total number of data points in the training data which was 127. [67]

$$f_{S}(x_{S})=\frac{1}{n}\Sigma_{i=1}^{n}f(x_{S},x_{C}^{\left(i\right)})$$ (1)

All data handling, visualizations, statistical analysis, ML modeling and interpretations were performed using R (V 4.0.3, Vienna, Austria). The data were recorded and secured in an encrypted database and were accessed only by the authors and the clinicians. The research study was conducted at a single rural community hospital and was approved by Montana State University Institute Review Board (approval number–BM050819 (EX))

## Results

### Model performance

**Fig 4:**
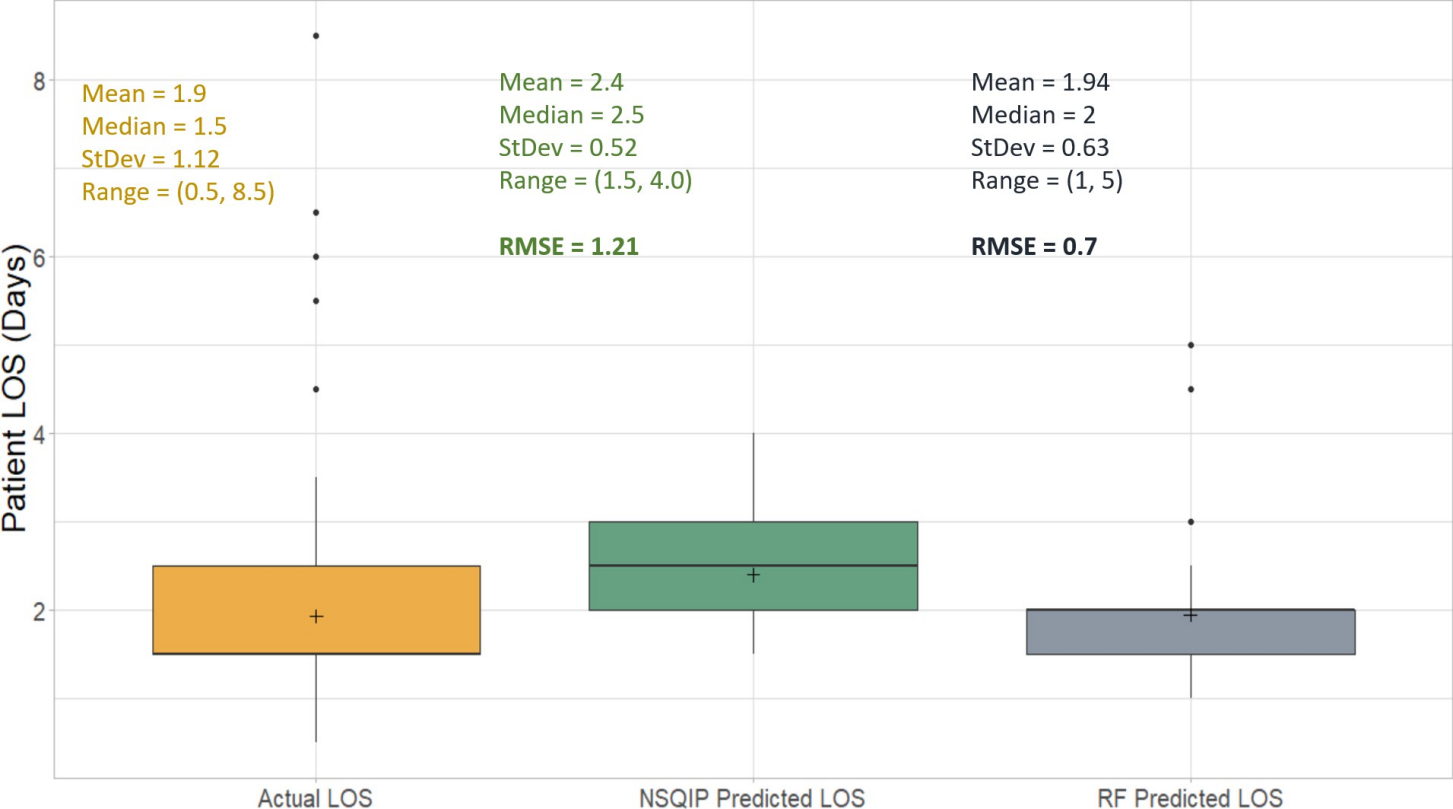
Comparison of NSQIP and Random Forest (RF).

**Fig 5:**
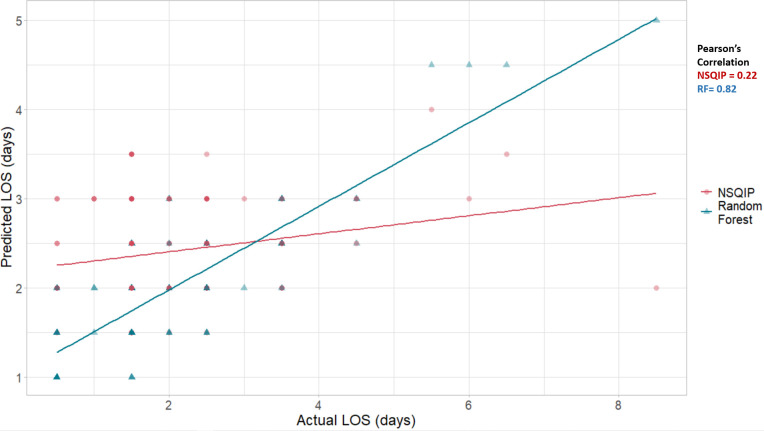
Pearson’s correlation for NSQIP and random forest.

The m_try_ with the lowest RMSE value was found at 2. The RMSE of RF for the train data (n = 127) and test data (n = 31) were 0.71 and 0.61, respectively. The RMSE of RF for the whole data (n = 158) was 0.7, which was lower than NSQIP which was 1.21 (Fig 4). The Pearson’s correlation between predicted and actual for NSQIP and RF were 0.22 and 0.82 (Fig 5). Compared to NSQIP, the RF model had lower RMSE and higher Pearson’s correlation, making it a better model for predicting patient LOS after the TJR procedure.

### Model interpretation

**Fig 6:**
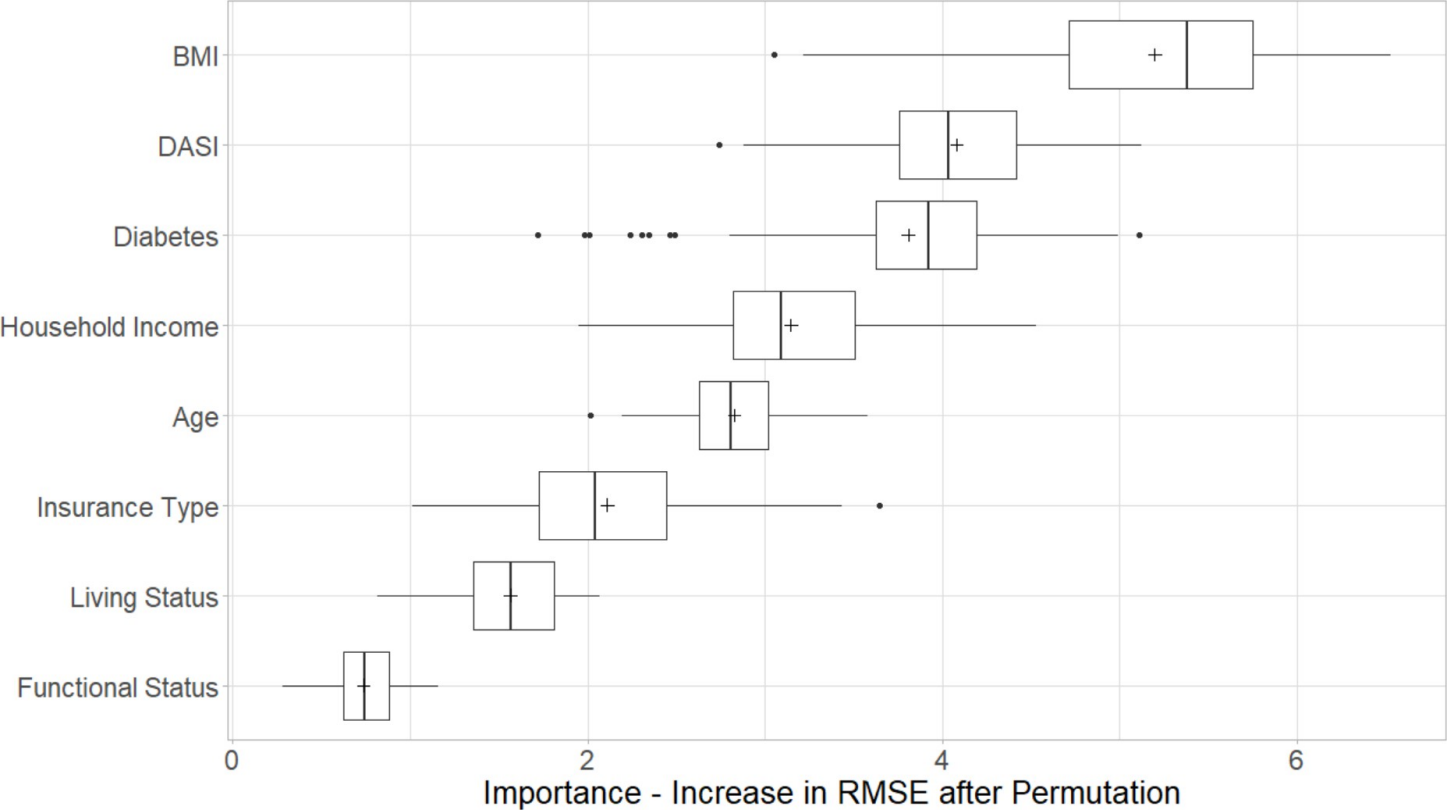
Variable importance using Permuted Feature Importance (PFI) method.

**Fig 7:**
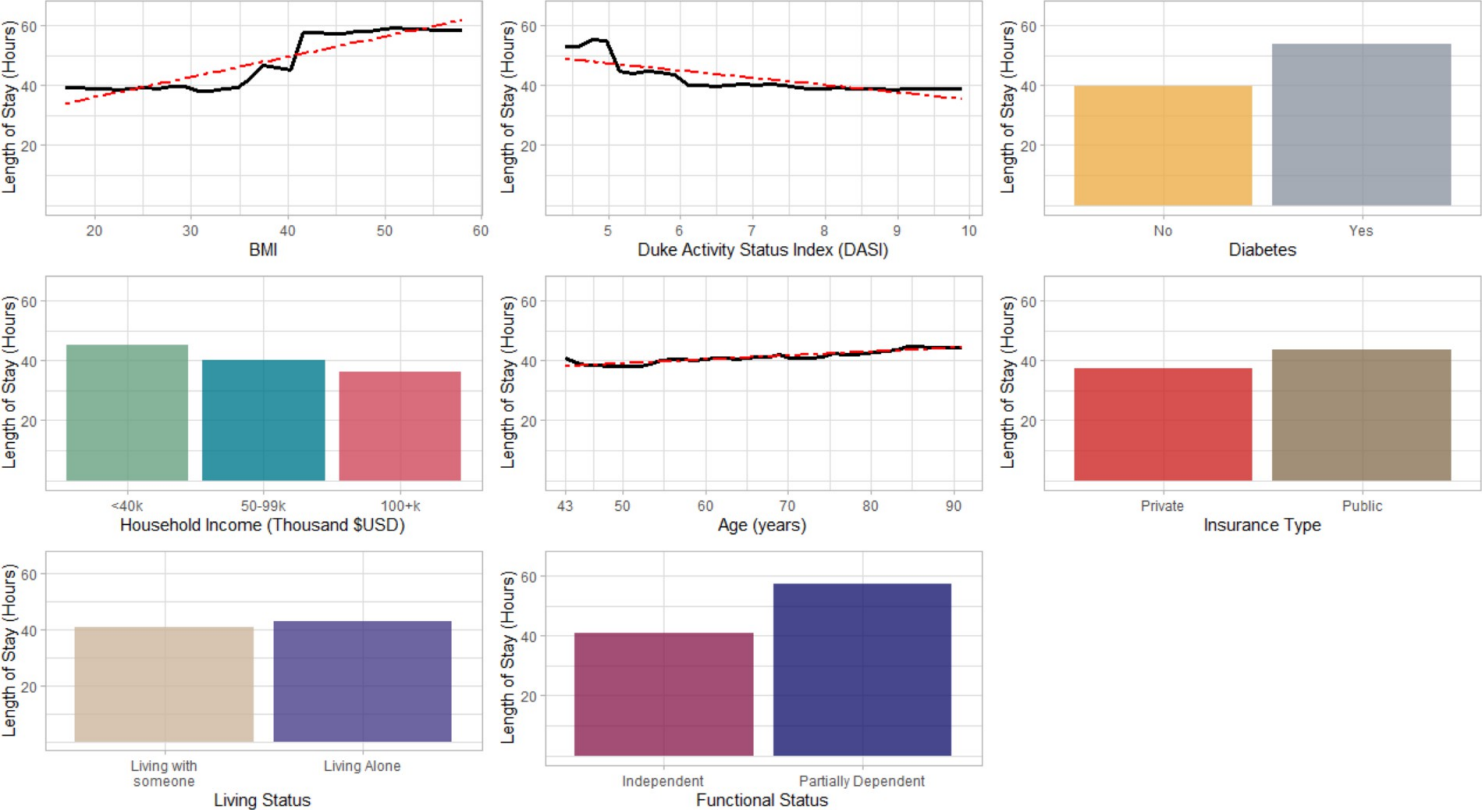
Partial Dependence Plot of independent variables against Length of Stay (LOS).

The PFI ranked independent variables based on their contributions to an accurate estimation of LOS using the RF training model. For example, BMI contributes most towards accurate prediction of LOS such that, if the values in BMI were randomly permuted, there will be an increase in overall RMSE by 5.1 (Fig 6). Similarly, the variables diabetes, DASI, living status, household income, ASA class, age, insurance type, and functional status were ranked most important to least important based on their average increase in RMSE after permutation (Fig 6). The PDP plots show the relationship between independent variables and the response variable (LOS) (Fig 7). More detailed explanation on their relationship is delineated in the discussion section of this paper.

## Discussion

The pilot study developed an ML model to predict LOS for TJR procedures that were performed at a small-scale community hospital (bed size less than 100) located in a rural area. The developed model predicted LOS (RMSE = 0.7) more accurately than the NSQIP risk calculator (RMSE = 1.21). The NSQIP was developed using a cohort of 1.4 million patient data, which were taken from 393 health institutions in the US [39]. Sixty-nine percent of the cohort were collected from a teaching or academic affiliation and 43% of the cohort were collected from large hospitals (i.e., bed size more than 500) [39, 73]. The inaccuracy in patient risk assessment is due to the vast differences between NSQIP cohort and the population (collected from a single hospital) on which NSQIP is used [15]. For instance, the TJR patients collected from a rural community hospital may be vastly different from the NSQIP cohort that was used to build the risk calculator. Moreover, the NSQIP includes insufficient numbers of orthopedic data i.e., only 12% of orthopedic patient data contribute to the analysis [73]. These factors contribute to the NSQIP’s suboptimal performance when predicting LOS for TJR procedures especially those performed at a community hospital. Another difficulty to why NSQIP poorly predicted LOS was the adoption of the PSH system [36]. Past studies have demonstrated that the implementation of PSH has led to a reduction in hospital LOS for TJR procedures [36, 74, 75]. Thus, similar to another study, it was observed that the NSQIP predicted LOS much higher than the actual LOS for TJR procedures performed at the PSH implemented community hospital [8].

To the authors’ knowledge, this pilot study is the first of its kind to develop an ML model that exceeds NSQIP risk calculator in predicting a TJR surgical outcome at a community hospital and the first to predict rural patients only. The developed ML model also provides a clearer interpretation compared to the NSQIP risk calculator. The model agnostic methods, PFI and PDP plots, revealed important independent variables and their relationship with LOS. The PFI model agnostic method ranked independent variables that most contributed toward accurate prediction of LOS (Fig 5). Through this, clinicians can prioritize those factors they should address first and design a suitable intervention in the preoperative phase to optimize patients, given the severity of the condition, surgery timing, and comorbidities.

The PDP model agnostic method illustrated the relationship between the independent variables and the response variable (Fig 7). The PDP for BMI indicated that patients with higher BMI (specially more than 40 Kg/m^2^) were more likely to stay longer at the hospital after the surgery compared to patients with lower BMI [76, 77]. For DASI, the LOS was found decreasing with an increase in DASI score. DASI assesses patient’s heart condition and functional capacity using likert scale questionnaires on daily activities, personal care, and recreation [40]. Patients with a higher DASI score represent they are healthier and more active. Thus, in this study, it was reasonable to observe that patients with lower DASI scores (especially less than 5 METs) had longer LOS compared to patients with higher DASI scores. The PDP for diabetes showed that on average, patients with diabetes had 14 hours longer LOS compared to the patients with no diabetes [78]. The income levels in the PDP plot of household income revealed that patients with lower household income were more likely to have longer LOS than patients with higher household income (100k+). Patients with lower household income (<40K) often delay their pre-operative treatments or even postpone their surgical procedures due to their financial barriers and cost of undergoing TJR procedures. These delays increase the complications at the time of surgery requiring a longer LOS to stabilize them before discharge [42].

Concerning age, like most studies, it was observed that the LOS was higher with an increase in age [11, 79, 80]. The PDP plot for insurance type showed that on average, patients who had public insurance as their payer had six hours longer LOS than patients who had private insurance. The public insurance payers in this study were Medicare and Medicaid. Compared to private insurance patients, Medicare patients are older (aged more than 65) with increased chances of having one or two medical complications [42]. The Medicaid patients are US citizens or legal permanent residents who are mostly from a low-income background with certain disabilities, behavioral health problems, or other complications [81]. Therefore, patients with public insurance are often more medically complicated which results in them staying longer at the hospital [42].

For living status, patients who were living alone, on average stayed three hours longer than patients who were living with someone (spouse, friends, or family). This was because a majority of the patients who lived alone had lower social support causing mental health problems (such as loneliness and sadness) which influenced them to stay longer at the hospital after surgery [45]. Another reason was most patients who lived alone had to rely on the hospital to arrange for a ride before discharge. This often takes longer than patients who get picked up by their family or friends during the discharge. Finally, for functional status, despite the limited sample size in the partially dependent category (less than 3%, Table 2), it was observed that on average, partially dependent patients had 17 hours longer LOS than fully independent patients.

Akin to many studies, variables BMI [76, 77, 82], age [11, 79, 80, 83, 84], and insurance type [42, 83, 84] were found as important predictors in this study well. Conversely, unlike many studies, the variable DASI was used in this study and was found as an important predictor of LOS. The researchers have used DASI as a preoperative assessment metric to evaluate postoperative risks, especially in colorectal surgeries [85, 86]. To the authors’ knowledge, the DASI has not been used in the past TJR studies to predict or measure its association with LOS. It was also observed that the ASA had no relationship in predicting LOS in this study which in contrast, had a significant effect on LOS in other TJR studies [2, 76, 82]. Along with the ASA, other clinical variables such as smoking, depression, and hypertension that were found important in other studies did not contribute to predicting LOS in this study [11, 84]. Instead, demographic variables household income and living status were found more important. This could be due to the implementation of the PSH system which included preoperative assessment and patient education, that helped clinicians to optimize patients with higher ASA, smoking, and hypertension complications [37, 87]. Differences in demographics and factors related to rural Montana location could be another reason for finding demographic variables household income, living status more important than clinical variables ASA, smoking, and hypertension, for predicting LOS.

The need for prediction such as ML is ever more necessary in rural healthcare systems as they do not receive the same attention as urban areas [14]. This research addresses that gap by introducing ML at a rural community hospital and making the community hospital more independent instead of relying on publicly available models/methods. This pilot study is also unique by using global agnostic methods at the rural community hospital for interpretation instead of using traditional interpretable prediction models such as general linear models and decision tree [67]. Future research built from this pilot study should focus on predicting other surgical outcomes such as discharge disposition and 90-days readmission rate [4]. Also, with Medicare’s recent removal of TJR surgeries from the inpatient list, future research from this pilot study should focus on developing a LOS prediction model to determine “inpatient” vs “outpatient” status for TJRs performed at rural hospitals [12].

Limitations in this pilot study include using only the RF model for prediction. Further research is on the way to applying different ML models such as Neural Networks, Boosted Trees, and Support Vector Machine (SVM) to discover how well they perform on these surgical data sets compared to RF. Second, the study used only a five-month duration (from August 2020 to December 2020) data with a very small sample size for the ML modeling. Yearly data with a high sample size (n > 1000) could have accounted for a better prediction, validation, and more robust interpretation. Third, the study was retrospective which may contain data collection biases that could alter the results and key findings [88]. Fourth, the testing data contained only 31 patients. More testing and validation data is required to confirm the developed model’s validity. Finally, this study was performed at a community hospital located in a micro-statistical area (with a population size less than 50,000). The results from this pilot study may not be generalizable to more rural places (e.g., with a population of less than 10,000).

## Conclusion

Delivering quality surgical care to TJR patients is a challenge to many US hospitals located in rural areas. Predicting LOS in surgery is an important factor as it helps rural hospitals deliver quality surgical service, ensure patient safety, and plan for resources such as inpatient beds. This research explored how a publicly available model (NSQIP) was not an ideal model to predict LOS after a TJR procedure performed at PSH implemented community hospital. Instead, a customized machine learning model–random forest–delivered more accurate LOS predictions despite the limited data available in rural surgical systems. Further, the random forest model also provided a better interpretation by ranking the important independent variables and illustrating its relationship against LOS. The pilot study is first of its kind to use ML at PSH incorporated rural surgical system to predict patient LOS. Results from this pilot study will contribute to helping rural surgical care by reducing LOS while improving patient satisfaction.
